# A case of small intestinal malignant lymphoma associated with intestinal obstruction: A case report

**DOI:** 10.1016/j.ijscr.2025.112007

**Published:** 2025-10-03

**Authors:** Hiroyuki Fujimura, Atsushi Goto, Akiyoshi Tanaka, Sigeru Yoneshiro, Hiroshi Itoh, Taro Takami

**Affiliations:** a Department of Gastroenterology and Hepatology, Hagi Civil Hospital, 3460-3 Tubaki, Hagi, Yamaguchi, 758-0061, Japan; b Department of Gastroenterology and Hepatology, Yamaguchi University Graduate School of Medicine, 1-1-1 Minami-kogushi, Ube, Yamaguchi, 755-8505, Japan; c Department of Surgery, Hagi Civil Hospital, 3460-3 Tubaki, Hagi, Yamaguchi, 758-0061, Japan; d Department of Radiology, Hagi Civil Hospital, 3460-3 Tubaki, Hagi, Yamaguchi, 758-0061, Japan; e Department of Molecular Pathology, Yamaguchi University Graduate School of Medicine, 1-1-1 Minami-kogushi, Ube, Yamaguchi, 755-8505, Japan

**Keywords:** Small intestine malignant lymphoma, Intestinal obstruction, Diffuse large B-cell lymphoma

## Abstract

**Introduction:**

Small bowel obstruction (SBO) is a common surgical emergency, most frequently caused by postoperative adhesions. However, neoplastic etiologies, including malignant lymphoma, should also be considered in the differential diagnosis.

**Case presentation:**

A 78-year-old Japanese man with a history of recurrent SBO since 2021 presented with nausea and abdominal distention. He had previously undergone conservative treatment on seven occasions. Contrast-enhanced computed tomography revealed small bowel dilatation with segmental wall thickening and enhanced contrast effect, along with enlarged mesenteric lymph nodes. He was diagnosed with SBO and admitted for emergency treatment. An ileus tube was inserted, and contrast study on day 3 revealed a stricture in the ileum, which the tube could not pass. Adhesive ileus and small intestinal tumor were both considered. As conservative treatment was ineffective, laparoscopic-assisted segmental ileal resection was performed. Histopathological examination revealed diffuse large B-cell lymphoma. Postoperative recovery was uneventful, and he was discharged on postoperative day 21. Positron emission tomography/computed tomography showed FDG uptake in mesenteric lymph nodes (SUVmax 3.7), consistent with Lugano stage II disease. Due to advanced age and dementia, the patient did not undergo chemotherapy. He remains under surgical follow-up without disease progression one year postoperatively.

**Discussion:**

This case highlights that repeated SBO may not always be caused by adhesions alone, and intestinal lymphoma should be considered, especially when imaging reveals suspicious findings.

**Conclusions:**

In cases of recurrent SBO, small intestinal lymphoma should be included in the differential diagnosis, even in elderly patients with a history of abdominal surgery.

## Highlights

• Small intestinal malignant lymphoma presenting with bowel obstruction is rare.
• Recurrent adhesive ileus may conceal an underlying small intestinal tumor.
• CT findings such as bowel wall thickening and lymphadenopathy aid diagnosis.

## 1 Introduction and importance

Tumors of the small intestine are relatively rare, accounting for only 1–5 % of all gastrointestinal neoplasms [1]. Among these, primary malignant lymphoma of the small intestine represents approximately 30–40 % of cases [2]. However, the presentation of bowel obstruction is uncommon [3]. We report a rare case of primary small intestinal malignant lymphoma presenting with bowel obstruction, which was successfully managed by laparoscopic-assisted surgical resection. This work has been reported in line with the revised SCARE criteria, 2025 [4].

## 2 Case presentation

**Fig. 1:**
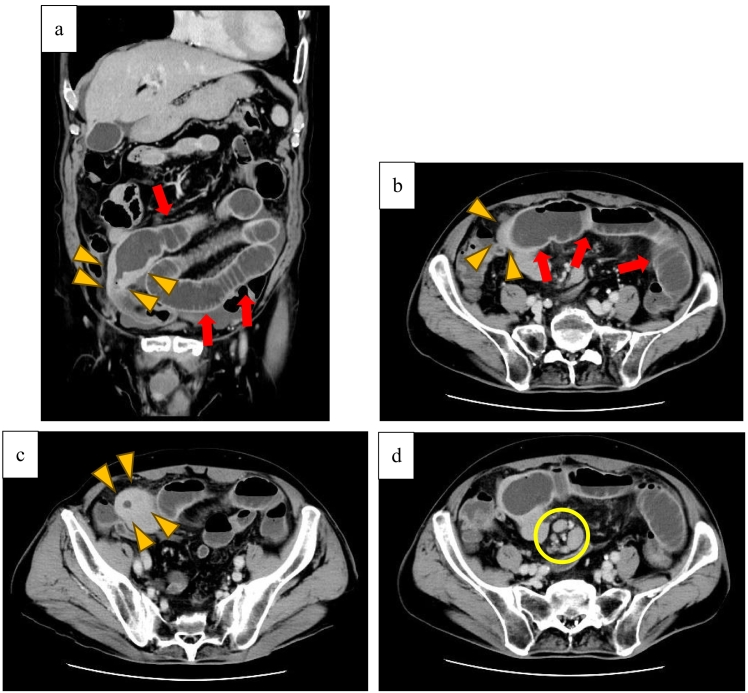
Contrast-enhanced CT revealed wall thickening of the small intestine (arrowhead) and marked bowel dilation and fluid retention on the oral side of the wall thickening (arrow). Enlarged mesenteric lymph nodes were also observed (circle). (a) Coronal CT image. (b)–(d) Axial CT images.

**Fig. 2:**
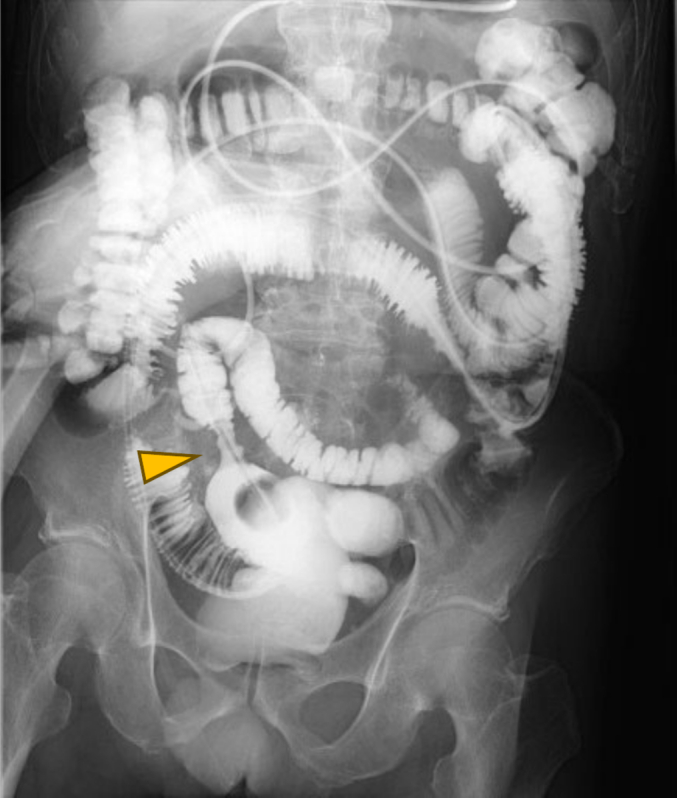
A contrast study through the tube showed a stricture in the ileum, which the balloon tip of the tube was unable to pass (arrowhead).

**Fig. 3:**
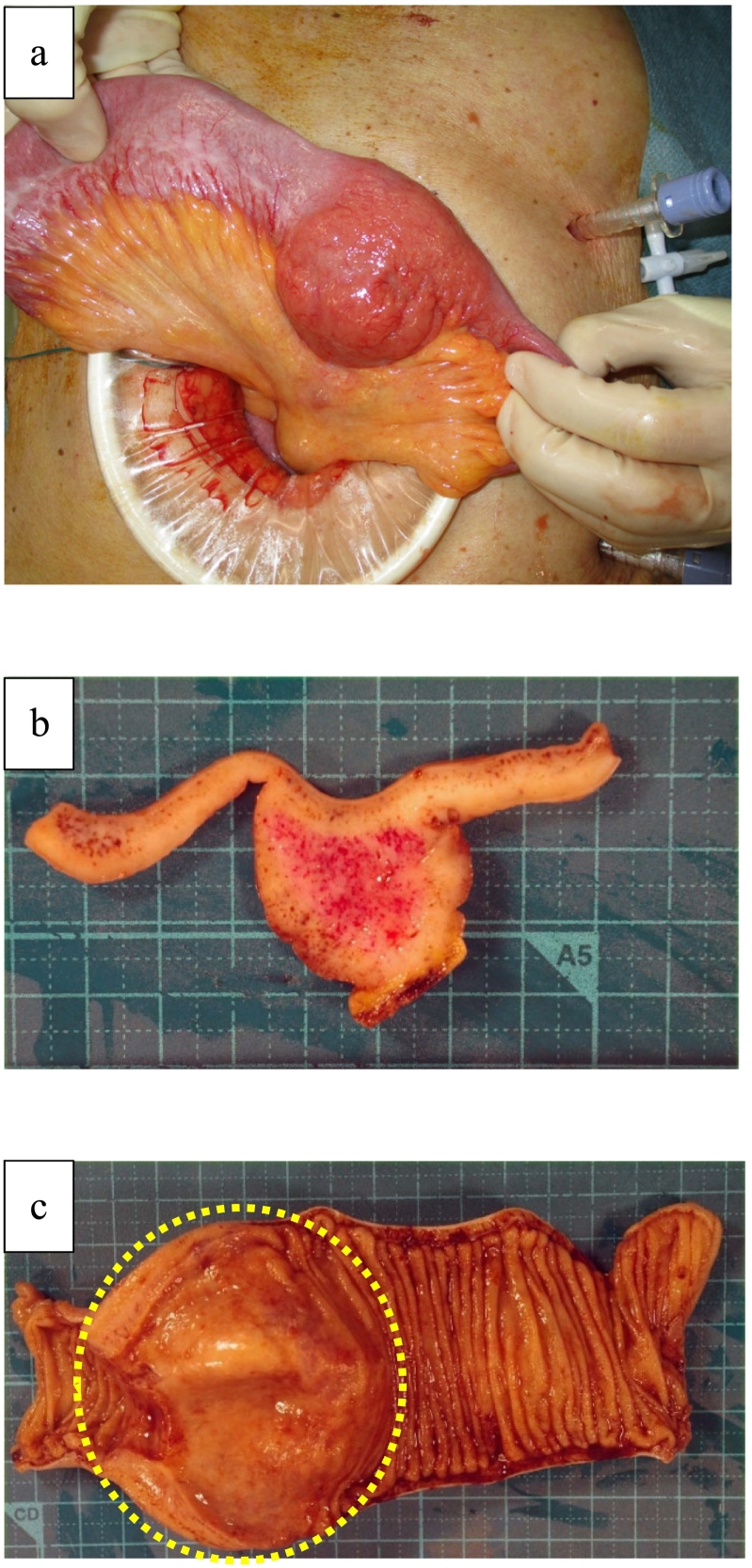
Intraoperative and gross pathological findings. (a), (b) A 3-cm mass protruding from the ileal wall was identified approximately 110 cm proximal to the ileocecal valve. (c) Although there was no obvious tumor exposure in the lumen, luminal narrowing was observed (circle).

**Fig. 4:**
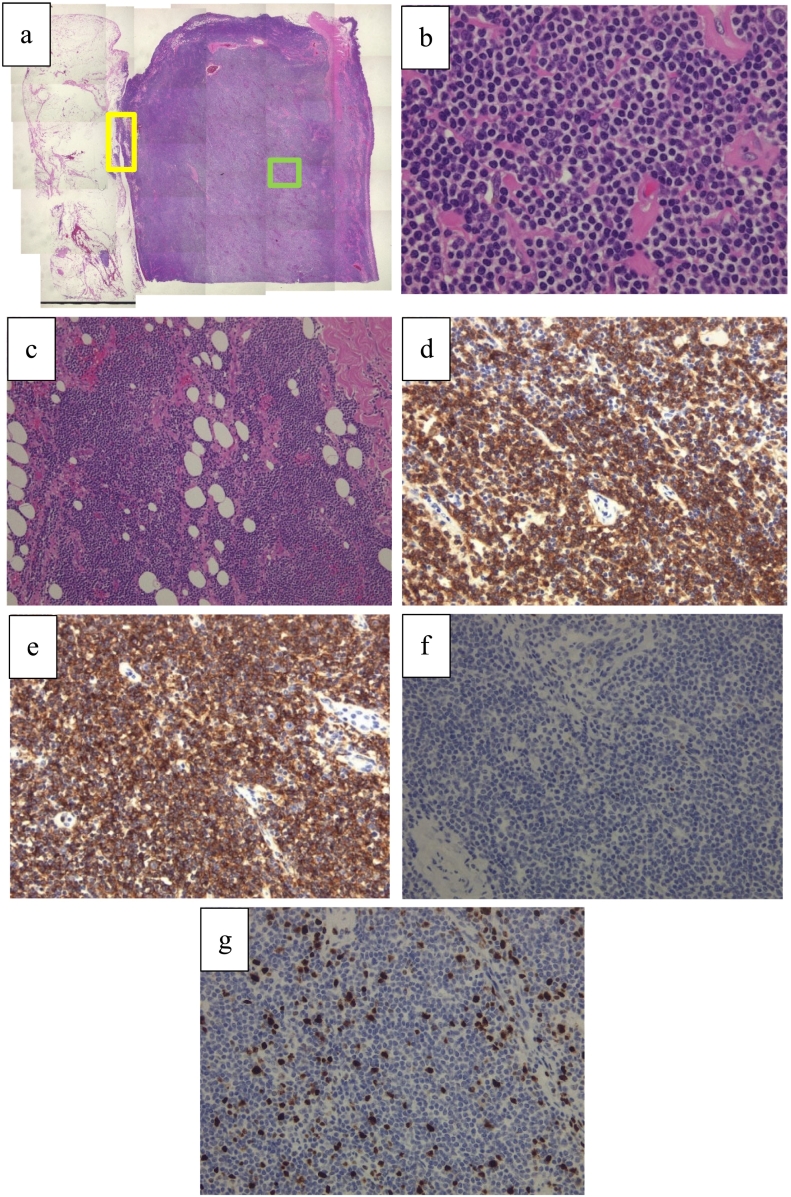
Histopathological findings. (a) Overall view (HE×10). (b) Enlarged image of Fig. 5 a (green square) (HE×200). The histopathological examination showed diffuse infiltration of large atypical lymphoid cells. (c) Enlarged image of Fig. 5 a (yellow square) (HE×40). Infiltration of atypical lymphocytes into the surrounding adipose tissue. (d) CD79a: positive (CD79a × 200). (e) CD20: positive (CD20 × 200). (f) Cyclin D1: negative (Cyclin D1 × 200). (g) The Ki-67 index: 20 % (Ki-67 × 200). (For interpretation of the references to color in this figure legend, the reader is referred to the web version of this article.)

The patient was a 78-year-old Japanese man who had experienced small bowel obstruction (SBO) seven times over the past three years. He presented to our hospital with complaints of nausea and abdominal distention. Plain abdominal radiography revealed dilated loops of the small intestine, with gas and air-fluid levels (niveau formation) observed within the abdomen. Contrast-enhanced computed tomography (CT) revealed wall thickening of the small intestine, proximal bowel dilatation, and enlarged mesenteric lymph nodes, leading to urgent admission with a diagnosis of intestinal obstruction (Fig. 1). The patient had a history of laparotomy, and the obstruction was suspected to be due to either adhesive ileus or a small intestinal tumor. The patient's serum tumor markers, including carcinoembryonic antigen (CEA) and carbohydrate antigen 19-9 (CA19-9), were within normal limits. The serum level of soluble interleukin-2 receptor (sIL-2R) was not measured. To relieve the obstruction, a long intestinal tube was inserted via an ultraslim transnasal endoscope (GIF-1200N; Olympus, Tokyo, Japan). Although decompression was successful, a contrast study through the tube on hospital day 3 revealed a stricture in the ileum, which prevented passage of the balloon tip (Fig. 2). As the stricture did not improve with continued conservative management, surgical intervention was planned. A laparoscopically assisted partial small bowel resection was performed. Widespread adhesions, considered to be a consequence of the prior operation, were identified. Intraoperatively, a 3-cm mass protruding from the ileal wall was identified approximately 110 cm proximal to the ileocecal valve (Fig. 3a). The umbilical incision was extended, and an approximately 15-cm segment of the ileum centered on the lesion was resected. Reconstruction was performed with end-to-end anastomosis. Gross examination of the resected specimen revealed a tumor protruding outward from the intestinal wall (Fig. 3b). Although there was no obvious tumor exposure in the lumen, luminal narrowing was observed (Fig. 3c). A histopathological examination revealed diffuse infiltration of large atypical lymphoid cells (Fig. 4a, b). Atypical lymphocytes were also observed infiltrating the surrounding adipose tissue (Fig. 4c). Immunohistochemistry was positive for CD79a and CD20 and negative for CD10 and cyclin D1 (Fig. 4c-f). The Ki-67 labeling index was approximately 20 % (Fig. 4g). These findings led to a diagnosis of diffuse large B-cell lymphoma (DLBCL) of germinal center B-cell origin.

The postoperative course was uneventful, and the patient was discharged on postoperative day 21. Positron emission tomography/computed tomography (PET/CT) performed after surgery revealed fluorodeoxyglucose (FDG) uptake in several enlarged mesenteric lymph nodes (SUVmax 3.7). The disease was classified as Lugano stage II and was considered to be low risk according to the International Prognostic Index for non-Hodgkin lymphoma. Considering the patient's advanced age and dementia, postoperative chemotherapy was not administered. The patient has been under surgical follow-up for one year and remains alive without evidence of disease progression or recurrence of intestinal obstruction.

## 3 Discussion

This case offers several important lessons in evaluating small intestinal tumors in patients presenting with bowel obstruction. First, recurrent SBO should not be attributed solely to adhesive ileus, especially in elderly patients with a history of prior abdominal surgery. Small intestinal tumors, including malignant lymphoma, should be considered for the differential diagnosis. Second, although SBO caused by stricture due to small intestinal malignant lymphoma is relatively uncommon, it remains an important etiology to consider. Finally, characteristic CT findings (e.g., circumferential wall thickening due to submucosal edema and mesenteric lymphadenopathy) can serve as valuable diagnostic clues.

Small intestinal malignant lymphoma is relatively rare, accounting for approximately 1–5 % of all gastrointestinal malignancies [1]. However, it constitutes 30–40 % of primary gastrointestinal lymphomas, second only to gastric lymphoma. The most common site of involvement is the ileum (60–65 %), followed by the jejunum (20–25 %) and duodenum (6–8 %) [5]. The disease can occur across a wide age range and shows a male predominance; the most common histological subtype is DLBCL [[6], [7], [8]]. The clinical presentation of small intestinal lymphoma is variable and nonspecific and includes abdominal pain, intussusception, gastrointestinal bleeding, or abdominal distension [9]. Even when they arise in the gastrointestinal tract, malignant lymphomas typically preserve intestinal wall distensibility because of their relatively soft consistency and minimal fibrotic component [10,11]. Therefore, SBO is considered uncommon in patients with malignant lymphoma. However, in this case, the reduced bowel wall flexibility caused by postoperative adhesions and extramural tumor growth appeared to have contributed to the development of intestinal obstruction.

There is currently no consensus on a standard treatment strategy for primary small intestinal DLBCL [12,13]. Treatment may include chemotherapy and/or radiotherapy, but in cases where there is a risk of obstruction or perforation, surgical resection is often performed as the initial treatment [14,15]. Even when surgery is indicated, postoperative chemotherapy is generally recommended, and minimally invasive surgery is preferred to facilitate early transition to systemic therapy [13]. The most commonly employed chemotherapy regimen is R-CHOP (rituximab, cyclophosphamide, doxorubicin, vincristine, and prednisone) [15,16]. However, in the present case, adjuvant therapy was withheld because of the patient's advanced age and dementia.

Instead, the patient was managed with close surveillance, including serial measurements of sIL-2R levels and contrast-enhanced CT imaging. No disease progression was observed for more than one year following surgery.

CT imaging is a key modality in the diagnosis of small intestinal lymphoma. Typical findings include segmental wall thickening, mesenteric lymphadenopathy, and infiltration of adjacent fat tissue [11]. In clinical practice, SBO in patients with prior abdominal surgery is frequently presumed to be adhesive in origin [17,18]. However, when imaging reveals unusual features, such as those noted in this case, malignant lymphoma should be considered in the differential diagnosis.

**Fig. 5:**
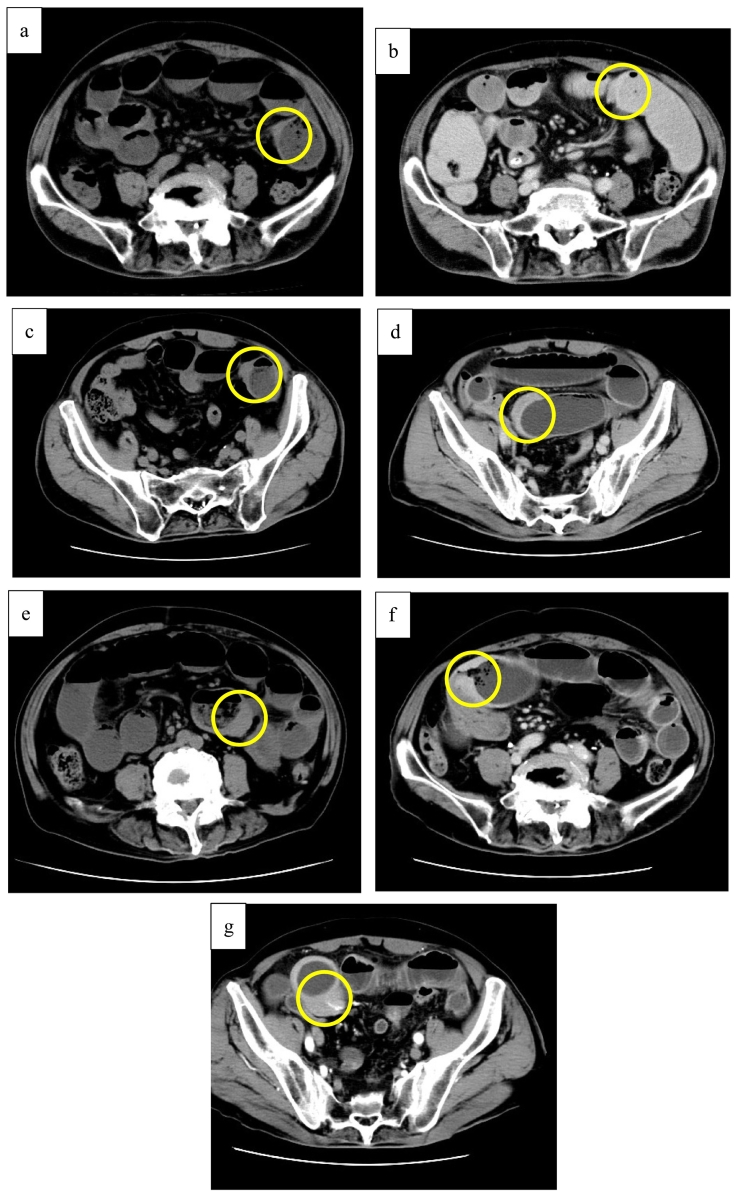
CT images. Retrospective evaluation of the tumor's chronological changes. Tumor locations are indicated by circles. (a) 2021 (b), (c) 2022, (d), (e) 2023, (f), (g) 2024.

CT images showing the seven prior episodes of intestinal obstruction are shown in Fig. 5 (a–g: a, 2021; b, c, 2022; d, e, 2023; f, g, 2024). A retrospective review of the serial images revealed segmental wall thickening as early as 2022 (Fig. 5c), suggesting that the disease may have been latent since that time.

## 4 Conclusions

We reported a rare case of small intestinal DLBCL presenting with recurrent bowel obstruction. This case highlights the importance of considering not only adhesions but also small intestinal tumors in the differential diagnosis, as well as the value of CT imaging and timely surgical intervention.

## Author contribution

Hiroyuki Fujimura: Study concept, patient management, data curation, manuscript writing (original draft), review & editing, and guarantor.

Atsushi Goto: Manuscript editing, literature review, supervision.

Akiyoshi Tanaka: Surgical management, manuscript review.

Sigeru Yoneshiro: Radiological interpretation.

Hiroshi Itoh: Histopathological diagnosis.

Taro Takami: Supervision, final manuscript approval.

All authors read and approved the final manuscript. All authors meet the ICMJE authorship criteria.

## Informed consent

Written informed consent was obtained from the patient for publication of this case report and the accompanying images.

## Ethical approval

Ethical approval is not required for case reports in our institution.

## Guarantor

Hiroyuki Fujimura

## Research registration number

Not applicable.

## Funding

This research did not receive any specific grant from funding agencies in the public, commercial, or not-for-profit sectors.

## Declaration of competing interest

The authors declare that they have no conflicts of interest regarding the publication of this paper.
